# Giant mucinous liposarcoma of the abdominal cavity: A case report

**DOI:** 10.1097/MD.0000000000039282

**Published:** 2024-08-16

**Authors:** Yi-Ming Li, Hai-Hong Zhu, Xiang-Qian Wang, Meng-Zhen Shi, Chao-Liang ShangGuan

**Affiliations:** a The Graduate School, Qinghai University, Xining, PR China; b Qinghai Provincial People’s Hospital, Xining, PR China.

**Keywords:** immunohistochemistry, lipoblasts, mucinous liposarcoma

## Abstract

**Rationale:**

Mucinous liposarcoma myxoid liposarcoma is a malignant mucoid soft tissue tumor derived from undifferentiated stromal cells in perivascular, subbody cavity and intermuscular space, and composed of cells at different stages of differentiation from preadipocytes to mature cells. In rare cases, it may change from lipoma malignancy. The main manifestations is painless mass, relatively slow growth, the course can last decades, the prevalence of liposarcoma in the population is 14% to 18%, mainly in adults, male prevalence is higher than women, but not significant. The main good hair part is the thigh, have mucinous sex, high differentiation type, dedifferentiation type, polymorphic type. Clinical diagnosis is difficult, and there are no obvious symptoms in the early stage, so the diagnosis should be combined with B ultrasound, MRI, CT, and other auxiliary examinations. The gold standard is pathological examination. In December 2023, our department admitted a patient with a mucinous abdominal mass. The report is as follows.

**Patient concerns:**

Does liposarcoma metastasize? Is any chemotherapy required after surgery? Will it ever relapse in the future? What is the survival period after surgery?

**Diagnosis:**

Mucinous liposarcoma.

**Interventions:**

Surgical resection of the sarcoma.

**Results:**

The nodule sample was 33 * 28 * 13 cm, with complete capsule, gray and yellow sections, fine texture, soft, gray, red, grayish, and yellow mucoid nodules in some areas, and the maximum diameter of the nodules was 21cm. Immunohistochemistry was: CD34 (+), CDK 4 (+), CK (−), Desmin (weak +), Ki67 (index 5%), MDM 2 (−), p16 (weak +), S-100P (+), Vimentin (+), BCL-2 (+). He was also sent to the Department of Pathology of Peking Union Medical College Hospital for consultation with Professor Lu Zhaohui, whose consultation opinion was in line with myxoliposarcoma.

**Conclusion:**

Retroperitoneal liposarcoma is a common retroperitoneal tumor, but it is relatively rare in clinical practice; the overall morbidity is low, mainly manifested as abdominal pain and abdominal distension, abdominal distension, and a long course of disease; it is not sensitive to radiotherapy and chemotherapy, and should be closely follow up by CT examination to understand the recurrence and metastasis.

## 1. Introduction

**Figure 1.**
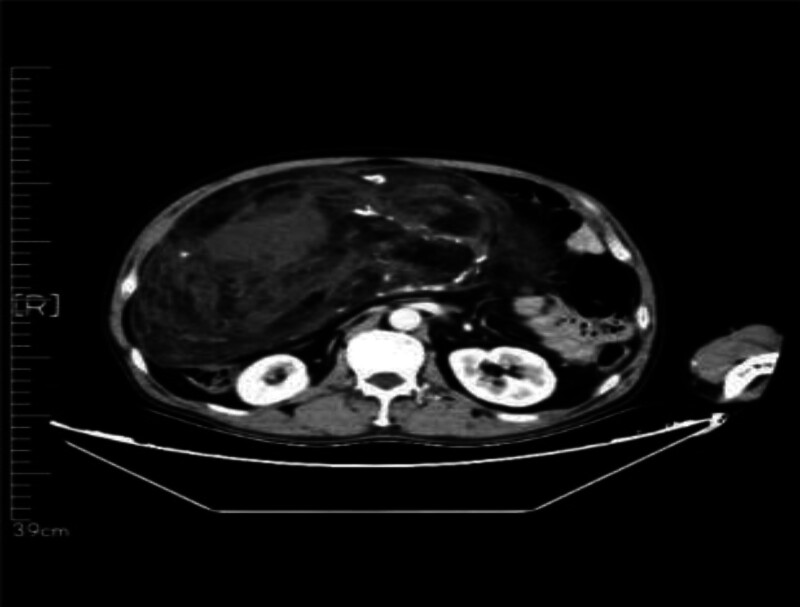
The whole abdominal plain scan was enhanced, and the abdominal–pelvic cavity was occupied by a huge mass.

**Figure 2.**
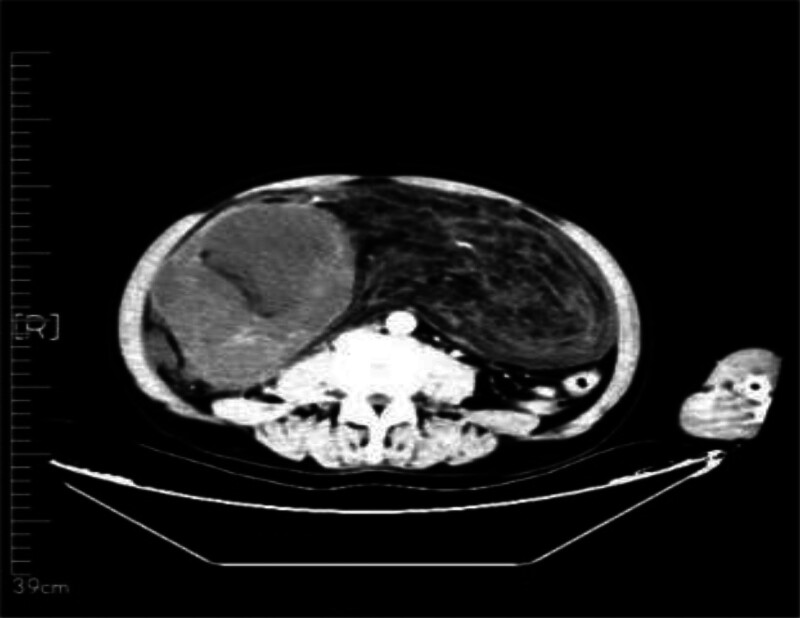
Obvious giant liposarcoma can be seen on CT.

The 57-year-old male patient was admitted to our department on December 11, 2023 due to “the examination found abdominal space-occupying lesions for more than 10 days.” The patient complained that the abdominal color ultrasound was checked in another hospital more than 1 month before the hospital, and then went to the “The First People’s Hospital of Haidong City” for treatment. The abdominal CT showed: the abdominal and pelvic capsule, liposarcoma? inflammatory myofibroblastoma? germinoma? The left periphery of the prostate had a cystic focus, and a degenerative cyst was considered, and MR was recommended. The patient had a previous history of “hypertension” for 2 years, up to 170/100 mm Hg. After spontaneous oral drug treatment (specific drug and measurement are unknown), the patient’s blood pressure was controlled between 120 and 140/80/80 and 90 mm Hg. In the previous year, “clavicle fracture” due to trauma was treated in another hospital. Physical examination: abdominal swelling, abdominal swelling can touch the medical large mass, hard quality, unclear boundary, tenderness (−), residual total abdomen without tenderness and rebound pain. After admission, on December 14, 2023, CT (Figs. 1 and 2): bronchitis; multiple small nodules in both lungs; emphysema; fiber proliferation with exudation; mediastinal lymph node calcification; left hilar calcifications; axillary lymph node enlargement; multiple old rib fractures; abdominal and pelvic space, possible liposarcoma. On CT imaging, multiple small cystic low density shadows in the left lobe of the liver, no enhancement in enhancement scan, huge space mass in the right abdomen, down to the pelvic cavity, large cross section of about 265 * 126cm, adipose tissue density and irregular thickening interval, moderate enhancement, multiple flocculent exudation of pelvic membrane, calcification and the gallbladder, pancreas, spleen and kidneys, morphology as usual, prostate size and morphology, no watery density in the pelvic cavity; no enlarged lymphadenopathy after the peritoneum;

## 2. Therapeutic process

**Figure 3.**
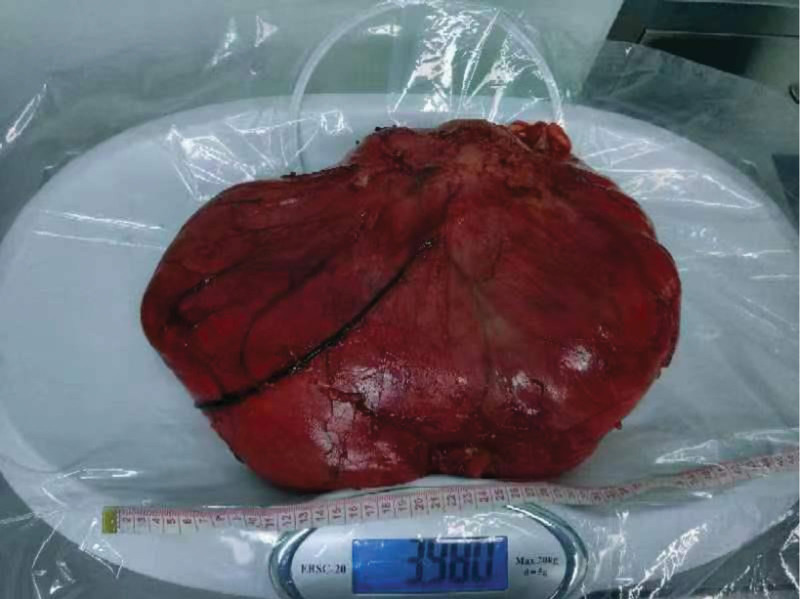
The general view of the patient during the operation, with a mass of up to 39.8 g and a diameter of 33 * 28 * 13 cm.

**Figure 4.**
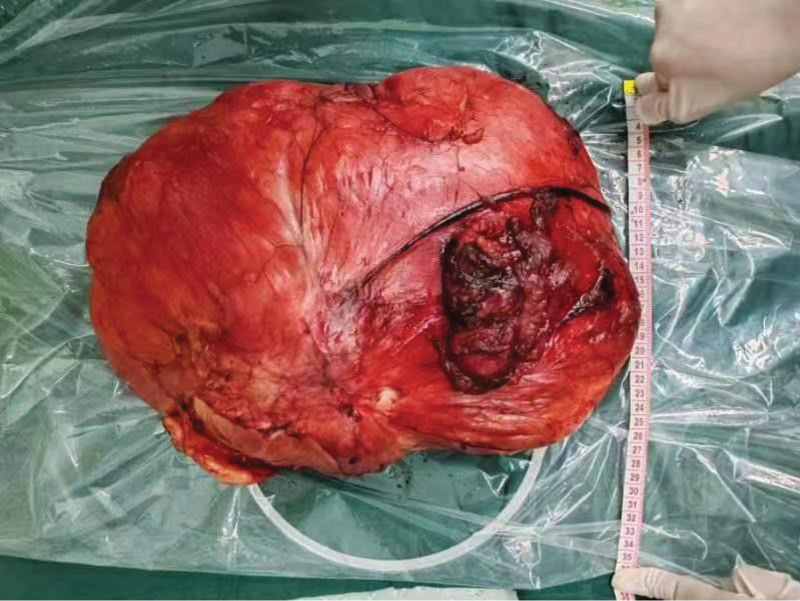
Visual observation of complete resection of liposarcoma in patients during surgery.

**Figure 5.**
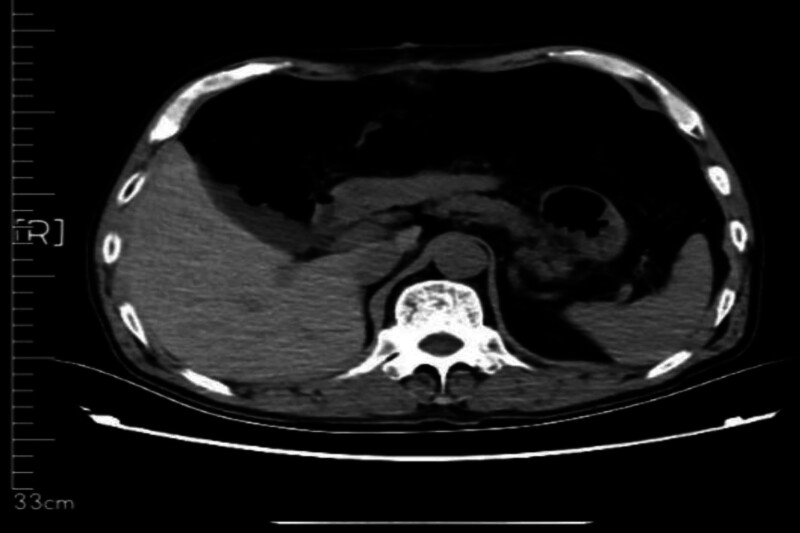
Subcutaneous exudation and air accumulation of the abdominal and pelvic wall after resection of a large retroperitoneal mass.

**Figure 6.**
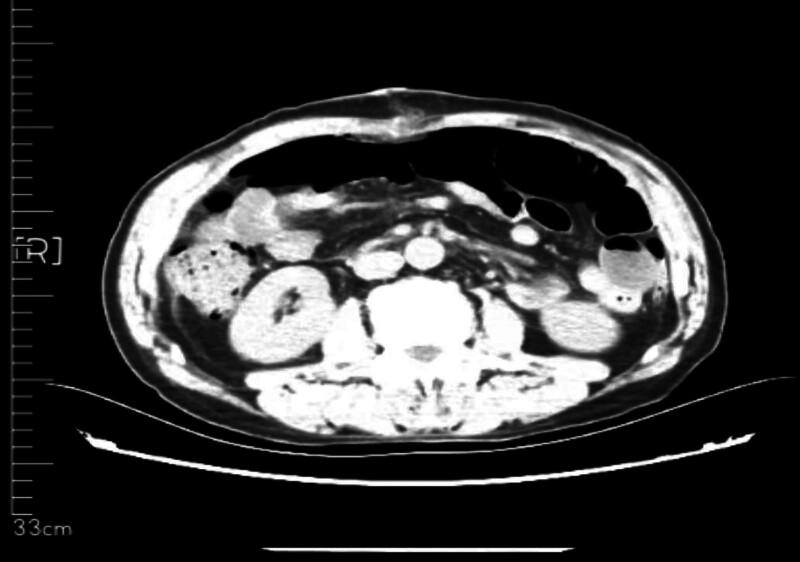
Postoperative CT shows that the liposarcoma in the abdominal cavity has been completely removed.

The patient’s vital signs was stable during the hospitalization, In general case, no fever, chills, nausea, no chest tightness, shortness of breath, no abdominal pain, no diarrhea, Therefore, after the inspection and perfection, On December 21, 2023, “retroperitoneal giant mass resection” under general anesthesia, Also informed the patient that due to the complicated condition, Major intraoperative bleeding, hemorrhagic shock may, unable to remove the clean, If surrounding organs such as gastrointestinal tract, adrenal gland, kidney, pancreas, urinary tract, need for an adrenalectomy, nephrectomy, etc., The specific surgical type should be decided according to the intraoperative situation, Does not rule out the possibility of intestinal fistula, pancreatic fistula, or ureteral reconstruction, After treating the urologist consultation, the urology department to assist the combined operation. During the operation, the median abdominal incision was about 35 cm long. The skin and subcutaneous tissue were cut open and the abdomen was entered layer by layer. Separation of abdominal adhesion zone and intestinal adhesion zone, explore abdominal cavity no obvious effusion, abdominal cavity visible a size about 42 * 20 * 20 cm size mass (Figs. 3 and 4), soft, poor mobility, along the mass was carefully free, from the ligament and adhesion, until the mass free to the right side peritoneal and bladder wall, combined with the preoperative data, the right mass differentiation degree, and the right mass tough, and lateral peritoneal and peritoneal adhesion serious, careful dissection, and avoid damage to the bladder and ureter. The abdominal mass was removed completely together, and part of the lateral peritoneal membrane was removed together, and the mass was removed completely. After the abdominal cavity was washed with warm saline, the bleeding was completely stopped. Due to the large mass and wide wound area, the peripheral ligament of the bladder was removed during the operation, and the bladder was suspended and the lateral peritoneum was repaired. Finally, one anti-adhesion irrigation solution was inserted in the abdominal cavity, and one latex drainage tube was placed in the pelvic cavity, and the right lower abdomen was closed layer by layer. After surgery, the resection specimen was sent for pathology, and the patient was admitted to the resuscitation room for resuscitation.The postoperative CT of the abdomen (Figs. 5 and 6) indicated that the resection had been clean.

## 3. Postoperative disease test results

On examination, the size of the nodules was 33 * 28 * 13 cm, the capsule was complete, the cut surface was gray and grayly yellow, fine texture and soft, some areas saw gray red, gray, and yellow mucoid nodules, and the maximum diameter of the nodules was 21 cm.

Immunohistochemistry was: CD34 (+), CDK 4 (+), CK (−), Desmin (weak +), Ki67 (index 5%), MDM 2 (−), p16 (weak +), S-100P (+), Vimentin (+), BCL-2 (+). He was sent to the Department of Pathology of Peking Union Medical College Hospital for Professor Lu Zhaohui for consultation, and the consultation opinion was in line with myxoliposarcoma.

## 4. Discussion

**Figure 7.**
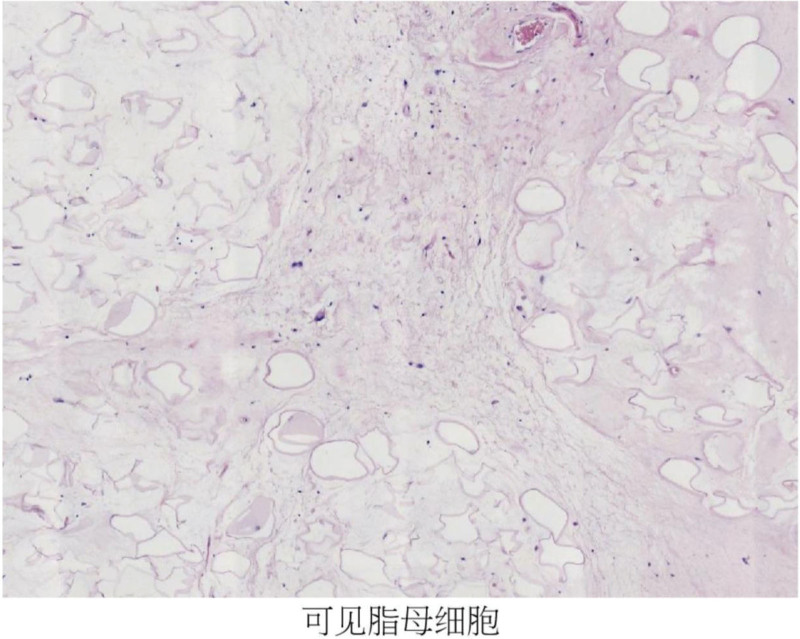
Typical lipoblasts are seen.

**Figure 8.**
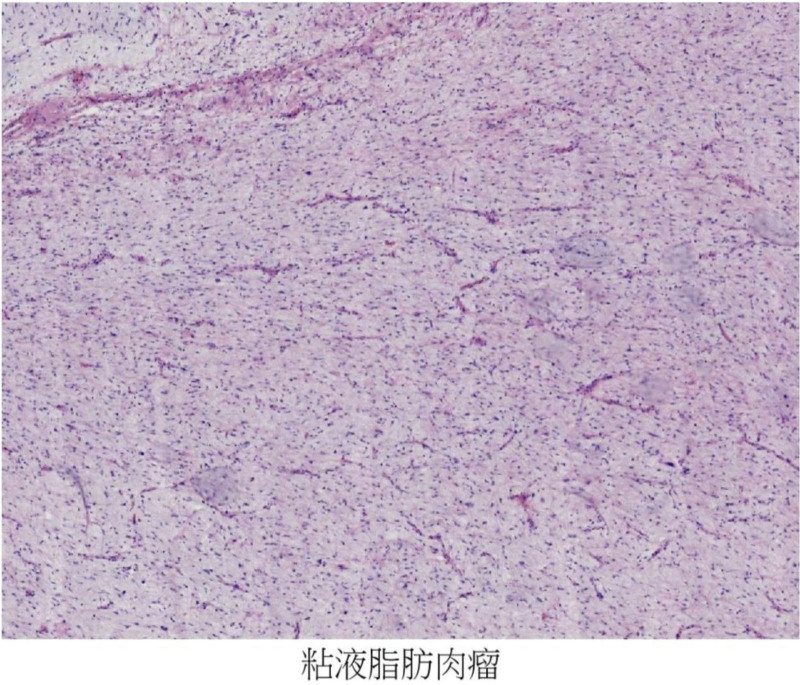
Mucinous liposarcoma is seen.

**Figure 9.**
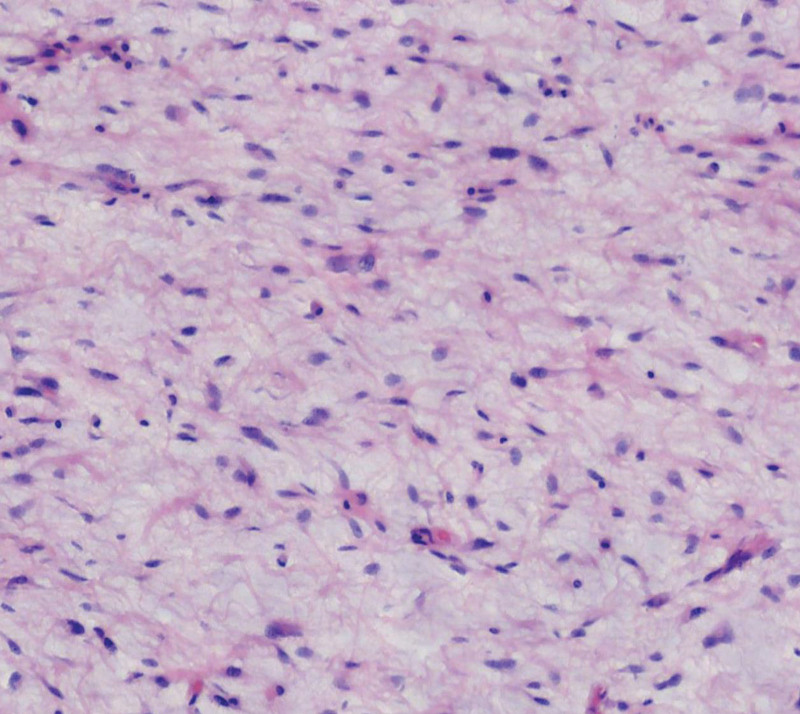
A mucoid matrix with a meticulous thin-walled capillary network.

**Figure 10.**
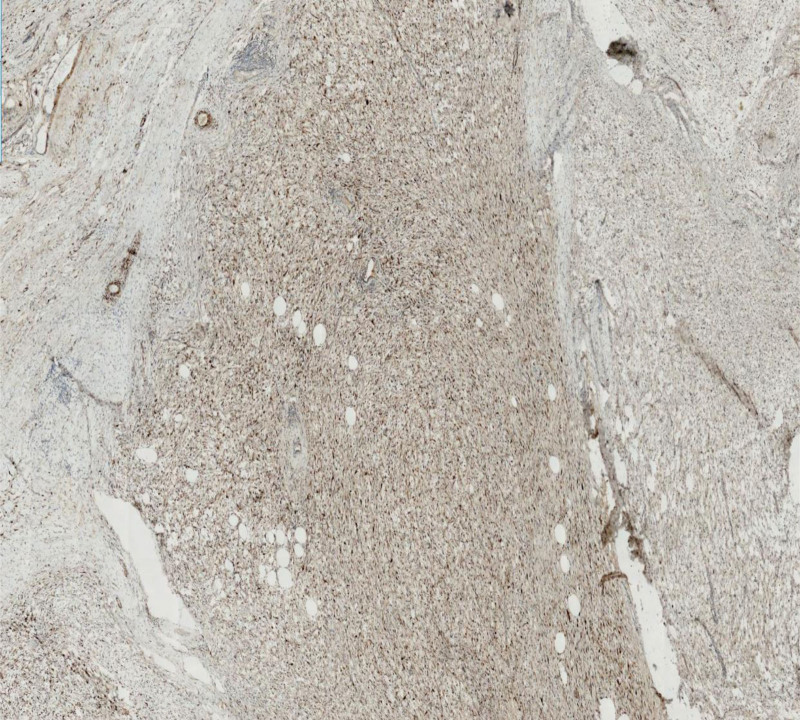
The visualization of Desmin (weak +) in this mucinous liposarcoma under HE staining and immunohistochemical microscope.

**Figure 11.**
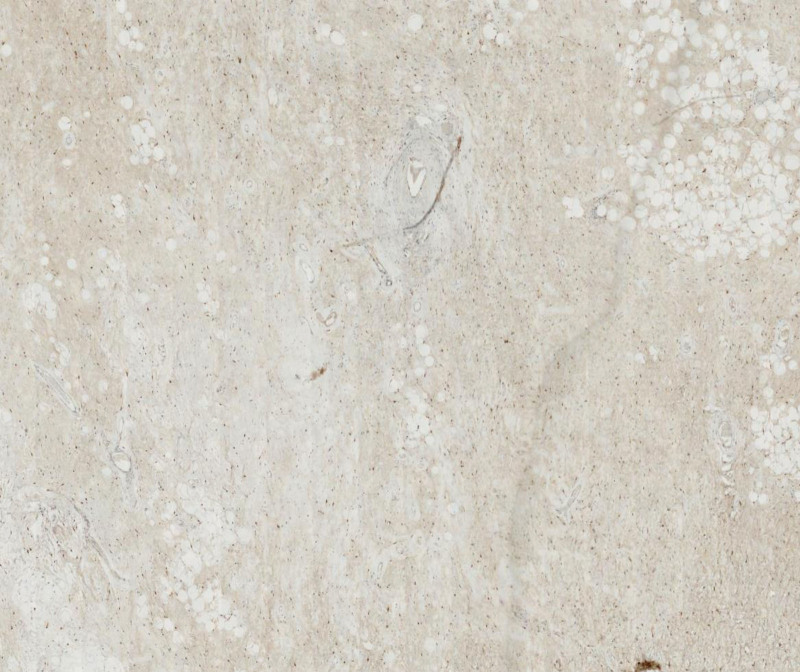
The visualization of CDK 4 (+) in this mucinous liposarcoma under HE staining and immunohistochemical microscope.

**Figure 12.**
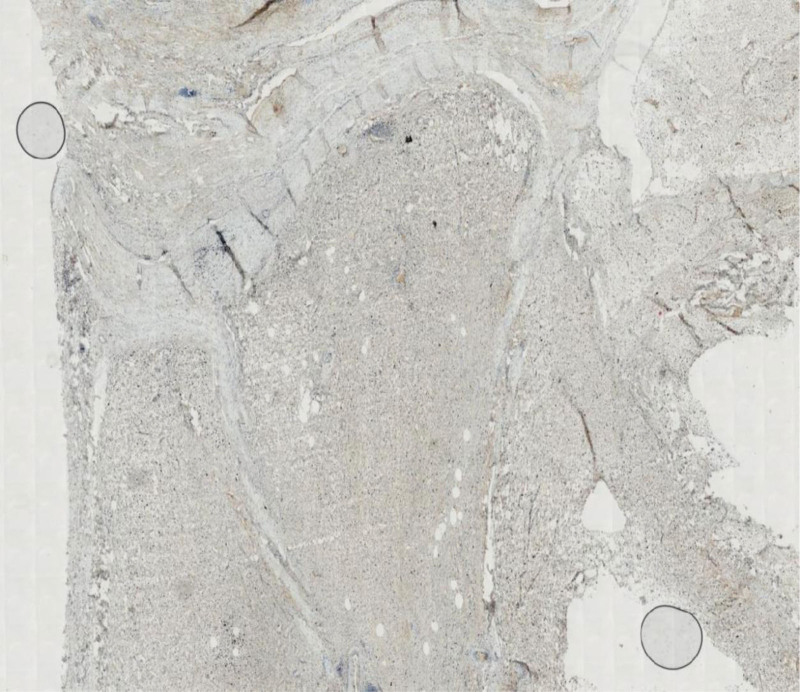
The visualization of Ki67 (index 5%) in this mucinous liposarcoma under HE staining and immunohistochemical microscope.

Retroperitoneal liposarcoma is the most common retroperitoneal tumor, but it is relatively frequently clinically rare, with low overall incidence,^[1,2]^ more common in adults and rarely in adolescents. In general, mostly nodular or lobulated, similar to lipoma, can also appear in myxoid or fish. A variety of tumor cells, characterized by the appearance of adipoblasts (the more obvious lipoblasts in this case Figs. 7 and 8), how many lipids of different sizes can be seen in the lipids, which can squeeze the cell nucleus and form a pressure trace. Retroperitoneal liposarcoma is deep in location, insidious in onset, and few patients have clinical manifestations, so it is difficult to clearly diagnose^[3]^ through clinical manifestations. Therefore, the tumor is often huge, and sometimes the tumor invades important blood vessels and organs, and the operation is more difficult, and even combined organ resection is needed. According to the World Health Organization classification of,^[4]^ liposarcomas are divided into 5 subtypes: highly differentiated liposarcoma/atypical lipomatoid-like tumors (well-differentiated liposarcoma/atypical lipomatous tumor, WDLPS/ALT), dedifferentiated liposarcoma (DDLPS), myxoid liposarcoma (MLPS), pleomorphic liposarcoma (PLPS), myxoid pleomorphic liposarcoma (MPLPS). In clinical practice, when encountering patients with liposarcoma, we often need to differentiate them. WDLPS usually has a larger tumor that is lobulated, and the pathological features can show relatively mature single running adipocytes. Under ultrasound, adipocytes, spindle cells, collagen cells, etc can be seen, and the echo inside the mass is uneven, with low or strong echogenic septa visible^[5]^; the pathological diagnosis of DDLPS requires the visualization of 2 components within the same tumor, namely well-differentiated liposarcoma and cell rich non lipogenic sarcoma. The boundary between the 2 is clear, and ultrasound shows multiple echoes within the mass, mainly strong and low echoes, with clear boundaries between each echo and regular morphology; PLPS presents as atypical adipocytes and tumor giant cells in pathology, with very few adipocytes showing mixed echoes of varying strength under ultrasound. The boundaries are clear, and the internal echoes are mainly medium to low, with uneven distribution of light spots and visible liquid dark areas. CT is the preferred examination method, and it has a high value in determining the type of tumor pathology, adjacent tissue infiltration, and malignancy degree.^[6]^ MRI is important to diagnose the site of origin, local range expansion and histological characteristics of liposarcoma, and in finding the invasion of the abdominal aorta or inferior vena cava.^[7,8]^ In this case of mucinous liposarcoma, not only can a varying number of adipoblastomas be seen under HE microscopy, but also distinctive mucinous like stroma accompanied by a fine thin-walled capillary network (Fig. 9) can be seen. In immunohistochemistry, Desmin (weak+) (Fig. 10), CDK4 (+) (Fig. 11), and Ki67 (index 5%) (Fig. 12) can be seen. Mucous liposarcoma can form t (12; 16) (q13; p11) translocations with each other in cellular genetics, and DDIT3 is a gene involved in fat differentiation. In addition, mucinous liposarcoma should also be differentiated from myxosarcoma, mucinous fibrosarcoma, synovial sarcoma, and schwannoma. In terms of surgical methods, due to the large volume and deep location of retroperitoneal liposarcoma, open surgery is usually adopted, but there are disadvantages such as large incision and serious tissue damage, and slow postoperative recovery. In recent years, laparoscopic technology has been developing continuously, providing a new strategy for the minimally invasive surgical treatment of liposarcoma.^[9,10]^ The transabdominal approach is superior to the retroperitoneal approach in both open and laparoscopic approaches. At present, the recommended standard treatment is radical resection of liposarcoma including adjacent organs.^[11,12]^ In this case, the peripheral ligament of the bladder was removed and the bladder was suspended and the lateral peritoneal was repaired. Gronchi et al^[13]^ showed that the recurrence rate of localized resection of liposarcoma at 5 years was 61%, and the recurrence rate of extended resection including adjacent affected organs was 36% (*P* = .007). Several factors are known to be associated with tumor recurrence or patient survival, such as age over 50 years, incomplete tumor resection, presence of implant and metastasis due to surgery, poor tumor differentiation and lymph node invasion and tumor volume, higher nuclear division index.^[14]^ Because liposarcoma often packages adjacent organs, it is insensitive to chemoradiation^[15,16]^, targeted drugs and immunotherapy are also not exact,^[17]^ so after radical resection, we recommend CT review every 3 months for the first 2 years,^[18]^ every 6 months for the 2 to 5 years, and annually thereafter, including MRI, ECT, PECT/CT if necessary.

## Author contributions

**Writing – original draft:** Yi-Ming Li.

**Writing – review & editing:** Hai-Hong Zhu, Xiang-Qian Wang, Meng-Zhen Shi, Chao-Liang Shangguan.
